# *SIAE* Rare Variants in Juvenile Idiopathic Arthritis and Primary Antibody Deficiencies

**DOI:** 10.1155/2017/1514294

**Published:** 2017-08-16

**Authors:** Eirini Sevdali, Elena Tsitsami, Maria Tsinti, Evangelia Farmaki, Efimia Papadopoulou-Alataki, Anastasios E. Germenis, Matthaios Speletas

**Affiliations:** ^1^Department of Immunology & Histocompatibility, Faculty of Medicine, School of Health Sciences, University of Thessaly, Biopolis 3, 415 00 Larissa, Greece; ^2^Pediatric Rheumatology Unit, 1st Department of Pediatrics, Children's Hospital “Aghia Sophia”, University of Athens, 115 27 Athens, Greece; ^3^First Department of Pediatrics, Hippokration General Hospital, Aristotle University of Thessaloniki, 546 42 Thessaloniki, Greece; ^4^Fourth Department of Pediatrics, General Regional Hospital Papageorgiou, Aristotle University of Thessaloniki, 564 03 Thessaloniki, Greece

## Abstract

Sialic acid acetylesterase (SIAE) deficiency was suggested to lower the levels of ligands for sialic acid-binding immunoglobulin-like receptors, decreasing the threshold for B-cell activation. In humans, studies of rare heterozygous loss-of-function mutations in *SIAE* gene in common autoimmune diseases, including juvenile idiopathic arthritis (JIA), yielded inconsistent results. Considering the distinct pathogenesis of the two main subtypes of JIA, autoinflammatory systemic (sJIA) and autoimmune oligo/polyarticular (aJIA), and a predisposition to autoimmunity displayed by patients and families with primary antibody deficiencies (PADs), the aim of our study was to analyze whether *SIAE* rare variants are associated with both the phenotype of JIA and the autoimmunity risk in families with PADs. A cohort of 69 patients with JIA, 117 healthy children, 54 patients, and family members with PADs were enrolled in the study. Three novel *SIAE* variants (p.Q343P, p.Y495X, and c.1320+33T>C) were found only in patients with aJIA but interestingly also in their healthy relatives without autoimmunity, while none of PAD patients or their relatives carried *SIAE* defects. Our results show that *SIAE* rare variants are not causative of autoimmunity as single defects.

## 1. Introduction

Autoimmune diseases represent a heterogeneous group of disorders characterized by hyperactive immune responses against “self,” potentially, due to the inability of the immune system to recognize self-antigens from pathogens. The majority of these diseases are non-Mendelian polygenic diseases, in which underlying complex genetic and gene regulatory mechanisms, in combination with environmental stimuli, lead to the emergence of autoimmunity [1, 2]. Genome-wide association studies have revealed a wide spectrum of common variants at hundreds of loci that contribute to autoimmune disease susceptibility; many of them are located into genes that are master regulators of immunological function ([1], reviewed in [3]). However, the effect size of these DNA polymorphisms is modest, as they account for relatively little disease risk and heritability; therefore, their contribution to developing autoimmunity is relatively low. It has been suggested that rare alleles of a large effect could account for what is called as “missing heritability,” out of the context of “common disease-common allele” hypothesis [4]. Nevertheless, Hunt et al. have shown recently that rare coding region variants have minor contribution in common autoimmune disease susceptibility, suggesting that multiple common variant signals of a weak effect could account for the missing heritability, instead of the rare ones [5].

Sialic acid acetylesterase (*SIAE*, Ensembl: ENSG00000110013) is an enzyme that regulates the cell surface acetylation of sialic acids. It has been proposed that SIAE functions in a B-cell intrinsic manner to prevent overactivation of B-cells by setting a threshold for B-cell receptor (BCR) signaling, contributing to the maintenance of immunological tolerance in mice [6, 7]. However, Mahajan et al. have recently revealed that the striking immunophenotype of SIAE-deficient mice that was described by Cariappa et al. [6] was attributed to a homozygous copy number variant in *Dock2* gene in a commercially available C57BL/6 mouse strain that has been used for backcrossing and that SIAE-deficient mice display no defects in B-cell development [8].

Nevertheless, rare heterozygous *SIAE* defects were first described by Surolia et al. as being enriched in patients with common autoimmune diseases; the majority of them constitute changes in highly evolutionary conserved sites, affecting protein catalytic function and *in vitro* secretion [9]. Analysis of the genetic locus of SIAE in 66,924 individuals of European ancestry failed to replicate the findings of Surolia et al. [9, 10]. Accordingly, several subsequent studies have reported varying results regarding the significance of *SIAE* locus in autoimmune disease susceptibility [11–16]. In this context, it has been suggested that further studies, including family-based association, should be applied in order to elucidate the precise role of rare *SIAE* variants in autoimmunity pathogenesis [9, 10].

Among the diseases analyzed in initial studies was juvenile idiopathic arthritis (JIA), the most common rheumatic disorder of childhood [9, 10], but no data regarding disease subtypes were provided. This is an important point, considering that JIA is characterized by clinically distinct manifestations, such as autoimmune oligoarticular JIA (oJIA) and polyarticular JIA (pJIA), which are the most commonly encountered forms (primarily oJIA), and the less common autoinflammatory systemic JIA (sJIA) [17]. Distinct pathogenic mechanisms are yielded behind the emergence of the different JIA phenotypes; oJIA and pJIA represent antigen-driven autoimmune diseases, as indicated by their positive associations with HLA genes, in contrast to the uncontrolled activation of the innate immune system, leading to the multisystem inflammation that characterizes sJIA (reviewed in [18]). Earlier genetic studies tended to group all children with JIA, while recent ones focus on the differences in JIA subtypes despite the inevitable reductions in sample size and subsequently the power of the study. However, the heterogeneity of the disease complicates the interpretation of the genetic data.

Moreover, autoimmune manifestations, including arthritis, represent one of the most severe complications in a proportion of patients with primary antibody deficiencies (PADs), which are associated with disturbed T- and B-cell homeostasis (reviewed in [19, 20]); however, it is still obscure whether autoimmunity and antibody deficiency share common genetic mechanisms. It has been reported that the presence of certain variants of immune genes, such as *TACI*, *CTLA4*, and *LRBA*, may be associated with the development of certain features of CVID [21]. It is also noteworthy that immunocompetent relatives of patients with PAD have an increased risk of developing autoimmunity ([22], reviewed in [23]).

Taking into consideration both the controversial association of rare *SIAE* variants with the emergence of JIA [9, 10] and the fact that *SIAE* locus has yet to be analyzed as a modifying genetic factor in PADs, the purpose of our study was to investigate the role of *SIAE* in the pathogenesis of these two multifactorial disorders. Moreover, due to the prevalence of autoimmunity in the family of patients with JIA and PADs, we analyzed the mutational status of *SIAE* in JIA and PADs relatives.

## 2. Materials and Methods

### 2.1. Subjects

Two cohorts of patients were enrolled in the study. The first consists of 69 patients with JIA (male/female: 22/47, mean age: 9.8 years, and range: 2.5–18.3) attending at the First Department of Pediatrics of Children's Hospital “Aghia Sophia,” University of Athens, Greece. Among them, 47 were diagnosed with oJIA (male/female: 11/36, mean age: 9.6 years, and range: 2.5–15.2), 11 with rheumatoid factor- (RF-) negative pJIA (male/female: 4/7, mean age: 10.7 years, and range: 2.9–18.3), and 11 with sJIA (male/female: 7/4, mean age: 9.9 years, and range: 5.1–15.4), according to the International League Against Rheumatism criteria [24]. One hundred and seventeen (117) children without autoimmunity (male/female: 36/81, mean age: 9.7 years, and range: 2.4–18.0), presenting to outpatient clinic for common disorders (flu, diarrhea, and so forth), have served as an age- and sex-matched control group and were analyzed for the incidence of the three novel *SIAE* mutations identified in patients with aJIA. Moreover, 10 relatives of patients with aJIA displaying rare *SIAE* variants, with/without autoimmunity (male/female: 6/4, mean age: 29.1 years, and range: 3–53), were also analyzed.

The second cohort of patients consists of 45 individuals with PADs (male/female: 24/21, mean age at diagnosis: 23.0 years, and range: 4–79); among them, 12 patients suffered from common variable immunodeficiency (CVID) (male/female: 7/5, mean age: 32.0 years, and range: 4–70) and 33 from IgA deficiency (IgAD) (5 of them with a coexistent IgG4D; male/female: 17/16, mean: 19.7 years, and range: 4–79). The diagnosis of PADs was based on the recent revised ESID criteria (http://esid.org/Working-Parties/Registry/Diagnosis-criteria), and the majority of patients suffered from autoimmune diseases. Nine (9) family members (male/female: 3/6, mean age: 50.0 years, and range: 17–85) of 4 CVID patients, with/without autoimmunity, were also analyzed for *SIAE* variants. The clinical and demographic data of all individuals enrolled in the study are presented in detail in Table 1.

The study has received ethical approval by the Institutional Review Board of the University Hospital of Larissa and the Children's Hospital “Aghia Sophia” of Athens, Greece. A written informed consent, prior to blood collection, was signed by all the participants as well as parents or relatives of patients where consent was not legally applicable (e.g., children). All experimental procedures performed were in accordance with the ethical standards of Helsinki declaration and its later amendments or comparable ethical standards.

### 2.2. Molecular Analyses

Peripheral blood samples were collected in K3-EDTA tubes from subjects during routine clinical visits; genomic DNA was isolated from samples using the QIAamp DNA Blood Mini Kit (Qiagen, Crawley, United Kingdom), according to manufacturers' instructions, and *SIAE* variants were detected by direct sequencing after polymerase chain reaction (PCR) amplification of all 10 exons and the exon-intron boundaries, as it has previously been described [9].

Furthermore, according to our findings, we studied the incidence of three novel *SIAE* variants detected in patients with the autoimmune types of JIA (p.Q343P, p.Y495X, and c.1320+33T>C), in their relatives and the healthy control group. Thus, for the detection of c.1320+33T>C and p.Q343P alterations, we set up PCR restriction fragment length polymorphism assay (RFLP) protocols. In particular, for the detection of p.Q343P mutation, a 360 bp fragment (encompassing exon 8) was amplified by PCR and subjected to *BtsCI* overnight digestion (New England Biolabs, Ipswich, UK) at 50°C. The presence of undigested PCR products was indicative of wild-type samples, whereas the presence of the mutation resulted in the digestion of the PCR products to 210 bp and 150 bp fragments.

For the detection of c.1320+33T>C genetic variant, a 433 bp fragment (encompassing exon 9) was amplified by PCR and subjected to digestion with TspDTI (EUR_x_, Gdańsk, Poland) at 70°C for 9 hours. Due to the presence of two *TspDTI* restriction sites, the wild-type PCR products were digested into 319, 89, and 25 bp fragments, while the presence of the mutated allele resulted in the loss of one restriction site and the identification of 2 fragments at 408 and 25 bp (homozygote) or 408, 319, 89, and 25 bp products (heterozygote). For the confirmation of the PCR-RFLP analysis results, the majority of PCR products of exons 9 and 30 randomly chosen PCR products of exon 8 were purified using a PCR purification system (Qiagen) and were directly sequenced using an ABI Prism 310 genetic analyzer (Applied Biosystems, Foster City, CA, USA) and a BigDye Terminator DNA sequencing kit (Applied Biosystems).

Finally, the detection of p.Y495X mutation was performed by direct sequencing after PCR amplification of exon 10 (as mentioned above), since the presence of the mutated or the wild-type allele does not create any restriction enzyme site.

### 2.3. Bioinformatic Analyses

In order to determine if the novel *SIAE* rare variants could affect the structure and function of the protein, 4 bioinformatic packages have been used, namely, SIFT (Sorting Intolerant From Tolerant, available at http://sift.jcvi.org/), Provean (Protein Variation Effect Analyzer, available at http://provean.jcvi.org/index.php), PolyPhen-2 (Polymorphism Phenotyping v2, available at http://genetics.bwh.harvard.edu/pph2/), and Mutation taster (available at http://www.mutationtaster.org/).

### 2.4. Statistical Analysis

Fisher's exact test (two-sided *p* values) has been used for the association analysis of the genotype frequency of novel *SIAE* variants between patients and controls. The above analysis was performed using the Statistical Package for the Social Sciences (SPSS) version 22 (IBM Corporation, NY).

## 3. Results and Discussion

**Figure 1:**
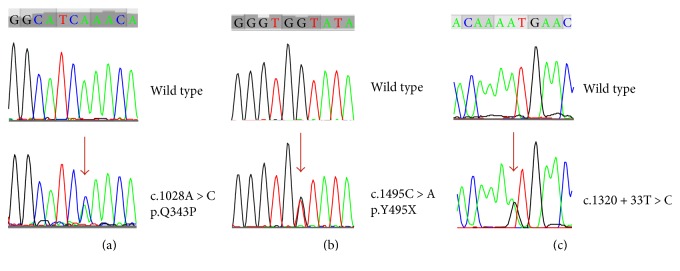
The novel *SIAE* rare variants identified in the patients of the study: (a) p.Q343P, (b) p.Y495X, and (c) c.1320+33T>C.

Three novel genetic *SIAE* variants were identified in the patients of the study, all in heterozygous state and only in patients with aJIA (Figure 1). The first alteration (c.1028A>C) was found in a patient with persistent antinuclear antibody- (ANA-) positive oJIA, resulting in the substitution of a neutral polar amino acid (glutamine) at the amino acid position 343 into a nonpolar amino acid (proline) (p.Q343P), within the catalytic portion of SIAE [25]. The second variant (c.1485C>A, p.Y495X) was a nonsense mutation in the C-terminal domain of SIAE, identified in a patient with RF-negative and ANA-positive pJIA. Finally, a novel genetic alteration located within intron 9 (c.1320+33T>C) of *SIAE* was found in a patient with persistent ANA-positive oJIA. Of note, these alterations were absent in patients with the autoinflammatory JIA subtype and in our healthy control group (odds ratio: 6.70, 95% confidence interval: 0.27–167.08, *p* = 0.312), as well as in all patients with PADs and their family members. Additionally, the examination of two public databases containing a detailed catalogue of variants derived from large-scale sequencing projects, the Exome Aggregation Consortium (available at http://exac.broadinstitute.org/) and the Exome Variant Server of the NHLBI GO Exome Sequencing Project (ESP) (available at http://evs.gs.washington.edu/EVS/), has shown that none of the novel mutations reported in this study have previously been identified.

The comparison of the nucleotide sequence of *SIAE* with nonredundant sequences present in NCBI using both the BLAST algorithm (available at https://blast.ncbi.nlm.nih.gov/Blast.cgi) and analysis with Mutation taster showed that the genetic alterations p.Q343P and p.Y495X involve residues that are conserved in serine hydrolases superfamily, in which SIAE belongs to, and are in consistence with the pattern of loss-of-function *SIAE* variants that have previously been described [9]. On the other hand, the intronic c.1320+33T>C alteration might also be damaging by affecting splicing, as supported by previous studies reporting the pathogenic significance of similar intronic defects (reviewed in [26]).

Bioinformatic tools can provide the predicted functional impact of coding variants supporting the interpretation of a possible consequence of a variant. In order to further elucidate the effect of the aforementioned alterations in protein function, we performed bioinformatic analysis, using 4 different tools, as mentioned above (Table 2). This type of analysis represents an alternative approach to identify whether a particular nucleotide and/or amino acid change results in profound structural alterations and, therefore, might influence functionally relevant parts of a protein. In this context, we identified that p.Q343P mutation involves a highly conserved residue and might result in splice site changes by strengthening existing splice sites (according to the Mutation Taster tool); such an event might result in the loss of N-glycosylation sites at 401 and 422 residues and subsequently affect the catalytic activity of the enzyme, as glycosylation has been suggested a prerequisite to give rise to the biologically active form of the enzyme [25]. Considering the nonsense mutation p.Y495X, it is located in a partly conserved domain, despite the high degree of conservation of the 495 residue and results in a slightly truncated protein, with less than 10% of the full wild-type protein length being missing. The abovementioned mutation does not meet the criteria for a nonsense-mediated mRNA decay, as it does not follow the “55 boundary rule” [27, 28], since it is located within the last exon of *SIAE,* supporting its probable pathogenic role. Finally, considering the intronic alteration c.1320+33T>C, bioinformatic analyses indicated that it is probably not pathogenic (Table 2). Nevertheless, the results of bioinformatic analyses are indicative and further functional studies are necessary to provide conclusive remarks.

**Figure 2:**
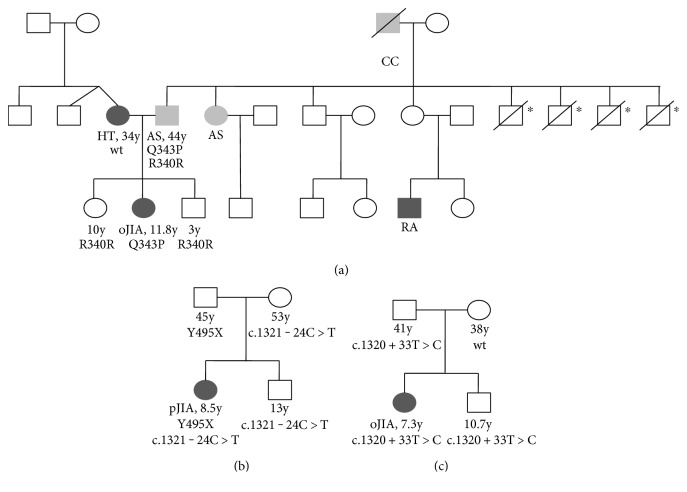
Pedigrees of patients with autoimmune juvenile arthritis and novel *SIAE* mutations. (a) Pedigree of the family with the p.Q343P variant; (b) pedigree of the family with the p.Y495X variant; (c) pedigree of the family with the c.1320+33T>C variant. Patients with autoimmunity are depicted in dark grey (HT, Hashimoto's thyroiditis; oJIA, oligoarticular JIA; pJIA, polyarticular JIA; and RA, rheumatoid arthritis). Patients suffering from other diseases are shown in light grey (AS, asthma; CC, colorectal carcinoma). All identified *SIAE* variants were in heterozygous state. The age of the individuals for whom genetic analysis was performed is also presented. ^∗^Individual II-11: death by car accident; individuals II-12, −13, and −14: death during childhood.

Furthermore, taking into consideration the high incidence of autoimmune diseases, mainly Hashimoto thyroiditis and rheumatoid arthritis, among first- and second-degree relatives of JIA patients [29], we investigated the prevalence of the novel variants in each respective patient's family (Figure 2). Interestingly, in the first family, the patient's father, diagnosed with asthma, was heterozygous for the p.Q343P variant, while the mother, diagnosed with Hashimoto's disease, along with all other healthy siblings analyzed, carried the wild-type alleles (Figure 2(a)). Similarly, the healthy proband's father carried the p.Y495X variant, while her mother and brother carried the wild-type alleles (Figure 2(b)); all individuals in this family did not suffer from autoimmunity. Finally, the healthy father and brother of the proband carrying the c.1320+33T>C alteration were also heterozygotes for this intronic variant (Figure 2(c)). All the aforementioned family members carrying the novel rare *SIAE* alterations were negative for ANA.

As presented in detail in Table 3, a number of previously described common single-nucleotide polymorphisms (SNPs) have also been identified in patients and controls of the study. The majority of them were silent polymorphisms (p.S156=, p.R340=, and p.T484=), and the p.T484= and p.S156= polymorphisms in our population are related (*χ*^2^ = 47.50, *p* < 0.001), which is in accordance with findings from the European population (LDlink, http://analysistools.nci.nih.gov/LDlink). Moreover, the catalytically intact *SIAE* variants p.K71R, p.M89 V, and p. A467V [14] were also identified in heterozygosity in both patients and controls (Table 3). It is noteworthy that the p.F404S mutation, a catalytically defective variant found both in a patient with JIA and in a relative with systemic lupus erythematosous but in none of 648 healthy subjects analyzed by Surolia et al. [9], was found in one individual of our healthy control group.

A strong genetic contribution may be more likely in JIA, as children have less time for environmental exposure that could influence disease risk. The strongest genetic contribution to the occurrence of aJIA has been attributed to the HLA locus, estimated to explain approximately 13% of the total variation in JIA susceptibility [30], confirming that there are still many non-HLA loci to be identified. Various SNPs in other genes of the immune system (i.e., *TRAF1-C5*, *VTCN1*, and *PTPN22*) have been revealed through genome-wide association studies (GWAS) as candidate JIA-predisposing loci (reviewed in [31]). However, common SNPs account for only one third of JIA susceptibility, rather higher than other complex autoimmune diseases, while the rest is attributed to rare variants, gene regulatory mechanisms, and gene-environment interactions (reviewed in [31, 32]). In this context, *SIAE* mutations may represent such rare defects that in concert with additional defects and/or interactions may contribute to the emergence of autoimmunity.

In regard with the analysis of *SIAE* in patients with PADs and their relatives, none of them was found to carry rare *SIAE* variants. Nevertheless, a number of known functionally intact *SIAE* variants (missense: p.G64S, p.K71R, and p.M89V and silent: p.L143=, p.S156=, and p.T484=) were identified in patients with PADs and their relatives with/without autoimmunity, without significant differences among disease subgroups (Table 4).

To the best of our knowledge, this is the first study investigating a possible contribution of the *SIAE* gene in the occurrence of autoimmunity in PADs. Autoimmunity represents one of the prevalent and most severe complications in CVID and IgAD, while a clustering of various autoimmune diseases has also been reported in the family environment of the affected patients [22, 23]. However, the genetic mechanisms behind the emergence of autoimmunity in these polygenic diseases are still not fully understood [19]. Taking into consideration the rare nature and heterogeneity of these disorders, we could not exclude a possible contribution of *SIAE* defects to the pathogenesis of autoimmunity in some patients with PADs. Therefore, the analysis of larger cohorts of such patients might be more determinant in confirming or not our results.

## 4. Conclusion

In this study, we identified novel *SIAE* mutations in patients with JIA; however, these rare variants were also present in patients' relatives without autoimmunity, supporting the notion that *SIAE* rare variants might have variable penetrance in the emergence of autoimmunity. On the other hand, we did not find rare *SIAE* alterations either in patients with PADs or in their relatives with autoimmunity. Thus, we conclude that rare *SIAE* variants do not result in overt autoimmunity as single defects. However, we cannot exclude the possibility that such variants might have an additive effect with other autoimmune disease risk loci. At the end, it is also clear that in the case of high heritability complex disorders, as autoimmune diseases, a number of clinically well-defined patients, families, and controls are required in order to draw reliable conclusions.

## Figures and Tables

**Table 1:** Clinical and demographic characteristics of the patients and controls of the study.

	JIA			Controls	PADs			PAD family members
	Total	aJIA	sJIA		Total	CVID	IgAD	
Number	69	58	11	117	45	12	33	9
Male/female	22/47	15/43	7/4	36/81	24/21	7/5	17/16	3/6
Age (years) (mean, range)	9.8 (2.5–18.3)	9.8 (2.5–18.3)	9.9 (5.1–15.4)	9.7 (2.4–18.0)	23 (4.0–79.0)	32 (4.0–70.0)	19.7 (4.0–79.0)	50.0 (17.0–85.0)
Arthritis (*n*, %)^a^	69, 100	58, 100	11, 100	0, 0	7, 15.6	3, 25.0	4, 12.1	1, 11.1
Autoimmune manifestations (*n*, %)	58, 84.1	58, 100	0, 0	0, 0	28, 62.2	11, 91.7	17, 51.5	4, 44.4
ITP (*n*, %)					6, 13.3	3, 25.0	3, 9.1	0, 0
AHA (*n*, %)					2, 4.4	1, 8.3	1, 3.0	0, 0
Pernicious anemia (*n*, %)					2, 4.4	2, 16.7	0, 0	0, 0
Thyroid disease (*n*, %)					13, 28.9	6, 50.0	7, 21.2	1, 11.1
Vitiligo (*n*, %)					2, 4.4	1, 8.3	1, 3.0	0, 0
Diabetes mellitus (*n*, %)					7, 15.6	0, 0	7, 21.2	0, 0
Celiac disease (*n*, %)					3, 6.7	0, 0	3, 9.1	0, 0
Others (*n*, %)^b^					3, 6.7	0, 0	3, 9.1	2, 22.2

AHA: autoimmune hemolytic anemia; aJIA: autoimmune juvenile idiopathic arthritis; CVID: common variable immunodeficiency; JIA: juvenile idiopathic arthritis; IgAD: IgA deficiency; ITP, idiopathic thrombocytopenic purpura; PADs, primary antibody deficiencies; sJIA, systemic juvenile idiopathic arthritis. ^a^Arthritis is reported in each group regardless of the autoimmune background, ^b^including patients with lupus, catastrophic antiphospholipid syndrome, Raynaud's syndrome, and autoimmune neutropenia.

**Table 2:** Bioinformatic analysis of novel *SIAE* genetic variants in patients with autoimmune juvenile idiopathic arthritis.

									Genotypes^a^ (allelic frequency)^b^			
Number	Location	DNA numbering	cDNA numbering	Amino acid substitution	PolyPhen-2 (score, sensitivity, specificity)	SIFT (score)	PROVEAN (score)	Mutation taster (probability)	oJIA	pJIA	sJIA	Controls
1	Exon 8	g.41498A>C	c.1028A>C	Q343P	Probably damaging (1.000, 0.00, 1.00)	Damaging (0.00)	Deleterious (−5.483)	Disease causing (0.999)	0/1/46 (0.011)	0/0/11 (0.000)	0/0/11 (0.000)	0/0/117 (0.000)
2	Intron 9	g.42795T>C	c.1320+33T>C	—	N/A	N/A	N/A	Polymorphism (0.999)	0/1/46 (0.011)	0/0/11 (0.000)	0/0/11 (0.000)	0/0/117 (0.000)
3	Exon 10	g.44266C>A	c.1485C>A	Y495X	N/A	N/A	Deleterious (−7.200)	Disease causing (0.999)	0/0/47 (0.000)	0/1/10 (0.045)	0/0/11 (0.000)	0/0/117 (0.000)

^a^Genotype: homozygous/heterozygous/wild type. ^b^Allele frequency of all *SIAE* rare defects (in total) in patients with autoimmune JIA: 0.009. The numbering of cDNA and amino acids corresponds to *SIAE* transcript: ENST00000263593.7.

**Table 3:** Allelic frequencies and genotypes of common *SIAE* variants in JIA patients and controls.

						Autoimmune JIA					sJIA		Controls		Europeans (NCBI)
						Total	oJIA		pJIA						
Number	Location	DNA numbering	cDNA numbering	Amino acid substitution	rs	Allele frequency	Genotypes^a^	Allele frequency	Genotypes^a^	Allele frequency	Genotypes^a^	Allele frequency	Genotypes^a^	Allele frequency	Allele frequency
1	5′ UTR	g.7560T>G	c.-36T>G		rs555947628	0.009	0/1/46	0.011	0/0/11	0.000	0/0/11	0.000	0/0/117	0.000	N/A
2	5′ UTR	g.7585T>A	c.-11T>A		rs202021641	0.000	0/0/47	0.000	0/0/11	0.000	0/1/10	0.045	N/A	N/A	0.002
3	Exon 2	g.11927A>G	c.212A>G	K71R	rs12282107	0.000	0/0/47	0.000	0/0/11	0.000	0/1/10	0.045	N/A	N/A	0.007
4	Intron 2	g.11964G>A	c.229+20G>A		rs512225	0.233	3/18/26	0.255	0/3/8	0.136	0/5/6	0.227	N/A	N/A	0.251
5	Exon 3	g.20536A>G	c.265A>G	M89V	rs78778622	0.026	0/3/44	0.032	0/0/11	0.000	0/0/11	0.000	N/A	N/A	0.051
6	Exon 4	g.26573T>C	c.468T>C	S156S	rs1942663	0.017	0/1/46	0.011	0/1/10	0.045	0/1/10	0.045	N/A	N/A	0.024
7	Intron 5	g.31722C>T	c.722+37C>T		rs79300393	0.009	0/1/46	0.011	0/0/11	0.000	0/0/11	0.000	N/A	N/A	0.023
8	Intron 5	g.31750C>T	c.722+65C>T		rs149793694	0.009	0/1/46	0.011	0/0/11	0.000	0/0/11	0.000	N/A	N/A	N/A
9	Intron 7	g.33978C>T	c.966+39C>T		rs142737112	0.009	0/1/46	0.011	0/0/11	0.000	0/0/11	0.000	N/A	N/A	0.005
10	Exon 8	g.41490T>C	c.1020T>C	R340R	rs35451312	0.000	0/0/47	0.000	0/0/11	0.000	0/1/10	0.045	N/A	N/A	0.007
11	Intron 8	g.41675A>G	c.1124+81A>G		rs620499	0.069	0/8/39	0.085	0/0/11	0.000	0/1/10	0.045	N/A	N/A	N/A
12	Intron 8	g.42499C>T	c.1125-68C>T		rs77343428	0.017	0/1/46	0.011	0/1/10	0.045	0/1/10	0.045	0/4/103	0.023	Ν/Α
13	Intron 8	g.42501T>C	c.1125-66T>C		rs140288211	0.009	0/1/46	0.011	0/0/11	0.000	0/0/11	0.000	0/2/105	0.009	N/A
14	Exon 9	g.42653T>C	c.1211T>C	F404S	rs201877149	0.000	0/0/47	0.000	0/0/11	0.000	0/0/11	0.000	0/1/106	0.005	0.002
15	Intron 9	g.44078C>T	c.1321-24C>T		rs138194723	0.078	0/8/39	0.085	0/1/10	0.045	0/1/10	0.045	0/10/107	0.043	0.029
16	Exon 10	g.44181C>T	c.1400C>T	A467V	rs7941523	0.000	0/0/47	0.000	0/0/11	0.000	0/1/10	0.045	0/1/116	0.004	0.003
17	Exon 10	g.44233G>A	c.1452G>A	Τ484T	rs7941327	0.009	0/1/46	0.011	0/0/11	0.000	0/1/10	0.045	0/3/114	0.013	0.024
18	3′ UTR	g.44414G>C	c.61G>C		rs145715586	0.000	0/0/47	0.000	0/0/11	0.000	0/0/11	0.000	0/1/116	0.004	N/A

^a^Genotype: homozygous/heterozygous/wild type. The numbering of cDNA and amino acids corresponds to *SIAE* transcript: ENST00000263593.7.

**Table 4:** Genotypes and allelic frequency of common *SIAE* variants in patients with primary antibody deficiencies (PADs).

						CVID			IgAD			Family members			Europeans (NCBI)
							Allele frequency			Allele frequency			Allele frequency		Allele frequency
Number	Location	DNA numbering	cDNA numbering	Amino acid substitution	rs	Genotypes^a^	Total	With autoimmunity	Genotypes^a^	Total	With autoimmunity	Genotypes^a^	Total	With autoimmunity	
1	Exon 2	g.11905G>A	c.190G>A	G64S	rs76655561	0/0/12	0.000	0.000	0/1/32	0.015	0.000	0/0/9	0.000	0.000	0.003
2	Exon 2	g.11927A>G	c.212A>G	K71R	rs12282107	0/0/12	0.000	0.000	0/1/32	0.015	0.000	0/0/9	0.000	0.000	0.007
3	Intron 2	g.11964G>A	c.229+20G>A		rs512225	0/5/7	0.208	0.227	1/10/22	0.182	0.176	0/3/6	0.167	0.125	0.251
4	Exon 3	g.20536A>G	c.265A>G	M89V	rs78778622	0/1/11	0.042	0.045	0/2/31	0.030	0.029	0/1/8	0.056	0.125	0.051
5	Exon 4	g.26532T>C	c.427T>C	L143L	rs201552273	0/0/12	0.000	0.000	0/1/32	0.015	0.000	0/0/9	0.000	0.000	0.001
6	Exon 4	g.26573T>C	c.468T>C	S156S	rs1942663	0/0/12	0.000	0.000	0/2/31	0.030	0.029	0/0/9	0.000	0.000	0.024
7	Intron 5	g.31722C>T	c.722+37C>T		rs79300393	0/0/12	0.000	0.000	0/1/32	0.015	0.029	0/0/9	0.000	0.000	0.023
8	Intron 7	g.33978C>T	c.966+39C>T		rs142737112	0/0/12	0.000	0.000	0/1/32	0.015	0.029	0/0/9	0.000	0.000	0.005
9	Intron 8	g.41675A>G	c.1124+81A>G		rs620499	0/1/11	0.042	0.045	0/4/29	0.061	0.029	0/1/8	0.056	0.000	N/A
10	Intron 8	g.42499C>T	c.1125-68C>T		rs77343428	0/0/12	0.000	0.000	0/1/32	0.015	0.000	0/0/9	0.000	0.000	Ν/Α
11	Intron 9	g.44078C>T	c.1321-24C>T		rs138194723	0/1/11	0.042	0.045	0/0/33	0.000	0.000	0/1/8	0.056	0.125	0.029
12	Exon 10	g.44233G>A	c.1452G>A	Τ484T	rs7941327	0/0/12	0.000	0.000	0/2/31	0.030	0.029	0/0/9	0.000	0.000	0.024

^a^Genotypes: homozygous/heterozygous/wild type. The numbering of cDNA and amino acids corresponds to *SIAE* transcript: ENST00000263593.7.
